# Access and Attitudes to HPV Vaccination amongst Hard-To-Reach Populations in Kenya

**DOI:** 10.1371/journal.pone.0123701

**Published:** 2015-06-26

**Authors:** Deborah Watson-Jones, Nelly Mugo, Shelley Lees, Muthoni Mathai, Sophie Vusha, Gathari Ndirangu, David A. Ross

**Affiliations:** 1 London School of Hygiene & Tropical Medicine, Keppel Street, London, United Kingdom; 2 Mwanza Intervention Trials Unit, National Institute for Medical Research, Mwanza, Tanzania; 3 Kenya Medical Research Institute, Nairobi, Kenya; 4 University of Nairobi, Nairobi, Kenya; 5 Division of Reproductive Health, Ministry of Health, Nairobi, Kenya; State University of Maringá/Universidade Estadual de Maringá, BRAZIL

## Abstract

**Background:**

Sub-Saharan Africa bears the greatest burden of cervical cancer. Human papillomavirus (HPV) vaccination programmes to prevent the disease will need to reach vulnerable girls who may not be able access health and screening services in the future. We conducted formative research on facilitators and barriers to HPV vaccination and potential acceptability of a future HPV vaccination programme amongst girls living in hard-to-reach populations in Kenya.

**Methods:**

Stakeholder interviews with Ministry of Health staff explored barriers to and support for the uptake of HPV vaccination. A situation assessment was conducted to assess community services in Maasai nomadic pastoralist communities in Kajiado County and in Korogocho informal settlement in Nairobi city, followed by focus group discussions (n=14) and semi-structured interviews (n=28) with health workers, parents, youth, and community and religious leaders. These covered marriage, knowledge of cervical cancer and HPV, factors that might inhibit or support HPV vaccine uptake and intention to accept HPV vaccine if a programme was in place.

**Results:**

Reported challenges to an HPV vaccination programme included school absenteeism and drop-out, early age of sex and marriage, lack of parental support, population mobility and distance from services. Despite little prior knowledge of cervical cancer and HPV, communities were interested in receiving HPV vaccination. Adequate social mobilisation and school-based vaccination, supplemented by out-reach activities, were considered important facilitating factors to achieve high coverage. There was some support for a campaign approach to vaccine delivery.

**Conclusions:**

Given the high level of support for a vaccine against cervical cancer and the experience of reaching pastoralist and slum-dwellers for other immunizations, implementing an HPV vaccine programme should be feasible in such hard-to-reach communities. This may require additional delivery strategies in addition to the standard school-based delivery, with vaccine offered at multiple venues, potentially through a campaign approach.

## Introduction

Sub-Saharan has the highest rates of cervical cancer globally[1]. Successful delivery of human papillomavirus (HPV) vaccination to prevent the disease has been pilot tested in several sub-Saharan African countries including Uganda and Tanzania, where over 80% of eligible primary school girls received three doses of the vaccine[2,3]. Rwanda has also achieved high coverage with its national, primarily school-based, HPV vaccination programme[4]. However, without targeted and focused interventions for hard-to-reach populations, it is likely that many individuals who suffer the highest burden of cervical cancer and who have least access to cervical screening programmes will also fail to receive the benefits of HPV vaccination.

These hard-to-reach populations are likely to comprise girls who never go to school, who drop out of primary school early, or who are frequently absent from school. This includes many girls living in informal settlements (slums) in large cities. It is estimated that 32% of the world’s population resides in these settlements; in sub-Saharan Africa 72% of the urban population lives in slum communities[5]. Data from a number of countries demonstrate that vaccine coverage is consistently lower than the national average in slum communities. In Mumbai and in Bangladesh, infant vaccination coverage is only 48% and 54% among slum dwellers compared to the national average of 60% and 78%, respectively[6,7]. In Nairobi, it is estimated that child immunization coverage for children aged 12–23 months is 58% in Korogocho and Viwandani slums compared to 73% coverage for Nairobi city as a whole[8]. Similar observations are seen in pastoralists, who routinely migrate with livestock and have difficult accessing services routinely[9].

Kenya is proposing a national HPV vaccination programme of girls enrolled in standard 4 of primary school following a GAVI-supported demonstration project of HPV vaccine delivery to girls in Kitui County that started in May 2013. As Kenya has limited prior experience of adolescent vaccination, our study was conducted to inform HPV vaccine delivery strategies for girls living in hard-to-reach settings such as urban slums and nomadic pastoralist communities.

## Methods

In order to explore perceptions of HPV vaccination and potential HPV vaccine delivery strategies for hard-to-reach communities, semi-structured interviews (SSIs) were conducted in Nairobi with 13 key Ministry of Health (MOH) and Education (MOE) personnel participating in vaccination, school-based health and out-reach services in order to explore their knowledge and views on HPV vaccination in schools and ways to access out-of-school girls. Field visits took place between November 2012 and February 2013 in two hard-to-reach communities; Korogocho, an informal settlement (slum) in Nairobi city that neighbours Dandora rubbish dump, and in nomadic Maasai pastoralist communities in Mashuru division in Kajiado District, selected after district health care providers and local chiefs identified this region as having particularly low school attendance.

An initial situation assessment was conducted in each community to gather information on health facilities and other available services and to map community schools, churches, community-based and faith-based organisations. From meetings with community leaders and structured walks through the communities, establishments and organisations, education and youth support activities were noted, and information gathered on how girls could be mobilised and where they could meet the team. Attempts were made to examine school registers for attendence and absenteeism information.

Fourteen focus group discussions (FGDs) were conducted with community and religious leaders, teachers, parents, girls aged 11–13 years and boys aged 13–17 years (Table 1) in order to explore community perceptions and knowledge of HPV and cervical cancer, sexual debut amongst girls, and to understand potential barriers, facilitators, constraints and acceptability for a future HPV vaccination programme. Boys were included because they can influence girls’ uptake of health interventions. These FGDs were followed by 28 semi-structured interviews (SSIs) with representatives of the above groups who did not participate in the FGDs, as well as with two health workers (HWs) to gain more in-depth and individual views on the above issues (Table 1). FGDs and SSIs additionally explored school enrolment and attendance, migration patterns, initiation of sex and marriage among girls, views about age- and sex-specific vaccination for HPV, vaccine side effects and attitudes towards parental consent. HWs and community members were asked to identify suitable venues for vaccination delivery and strategies to maximize vaccine coverage. Teachers and youth FGD participants in both sites and religious leader FGD participants in Korogocho also participated in listing, ranking and scoring exercises to further explore potential barriers and facilitating factors related to a potential HPV vaccination programme, using adapted participatory learning and action (PLA) tools[10].

**Table 1:** Focus group discussions, semi-structured interviews and stakeholder interviews.

	Korogocho	No.	Kajiado	No.	Nairobi	No.
Focus Group Discussions (FGDs)	Girls 11–13 yr (N = 8)	1	Girls 11–13 yr (N = 8)	1		
	Boys 14–17 yr (N = 9)	1	Boys 14–17 yr (N = 8)	1		
	Parents	2	Parents	2		
	Mothers (N = 8)		Mothers (N = 9)			
	Fathers (N = 8)		Fathers (N = 8)			
	Teachers (N = 11)	1	Teachers (N = 9)	1		
	Community leaders (N = 9)	1	Community leaders (N = 8)	1		
	Religious leaders (N = 9)	1	Religious leaders (N = 9)	1		
Total FGDs		7		7		
Semi Structured Interviews (SSIs)	Girls	2	Girls	2		
	Boys	3	Boys	1		
	Parents	2	Parents	2		
	Teachers	2	Teachers	2		
	Community leaders	2	Community leaders	3		
	Religious leaders	2	Religious leaders	2		
	Health workers	1	Health workers	2		
Total SSIs		14		14		
Stakeholders SSIs General			Chief—Osilalei location, Mashuru, Kajiado County	1	Chief—Korogocho, Nairobi	1
Stakeholders SSIs—Health	Matron in charge Kariobangi Health Centre, Kasarani District, Nairobi (serves Korogocho)	1			Reproductive Health Advisor–Division of Reproductive Health	1
					District Medical Officer Kasarani District, Nairobi	1
					Programme Officer Division of Child health	1
					Head of Division of Vaccines	1
					Vaccine Stores Manager Nairobi	1
					Programme Officer Adolescent Health-Division of Child and Adolescent Health	1
					Deputy head-Division of Reproductive Health	1
Stakeholders SSIs—Education	Head teacher- Daniel Comboni Primary School, Korogocho,Nairobi	1	Head teacher-Megumi Primary School- MashuruKajiado	1		
			District Education Officer	1		
Total Stakeholders		2		3		8

Potential participants were identified through the assistance of community health workers (CHWs), local chief’s offices, and the Catholic Mission for girls working in Dandora dump. HWs were recruited through MOH facilities. Informed written consent was sought including permission to record the interviews using digital recorders. Participants less than 18 years old provided informed assent after their parents/guardians had consented for them to participate. Interviews were conducted in Swahili, Maasai or English depending on the respondent’s choice. Recordings were transcribed and Swahili and Maasai interviews were translated into English. The transcripts were imported into NVIVO version 9 (QSR International). An initial coding frame was developed and a framework analysis approach was used to code data, and themes were guided by the research questions. Quality checking was conducted by two social scientists.

The study was approved by the ethics committees of Kenyatta National Hospital/University of Nairobi and the London School of Hygiene & Tropical Medicine. Informed written or (witnessed consent with a signature from a witness if the person was illiterate)was obtained for parents or guardians on behalf of the minors/children enrolled in the study. Minors were asked to provide written informed assent. These consent procedures were also approved by the above ethics committees.

## Results

### Context for providing HPV vaccines in the study areas

#### Country experience in adolescent vaccination and in hard-to-reach communities

Experience with adolescent vaccination in the Division of Vaccines (DVI) of the MOH was primarily drawn from a recent tetanus toxoid (TT) vaccination programme which included girls aged 10 years. This had led to rumours that the vaccine had sterilising or contraceptive properties and these were particularly prevalent in coastal communities, propagated by some religious leaders. To improve acceptance, DVI eventually vaccinated both boys and girls in the coastal region. To reach hard-to-reach communities, the TT vaccination programme used defaulter tracing by CHWs, social mapping to identify pastoralist movement patterns and establishment of mobile clinics to provide vaccinations where necessary. TT vaccination via mobile teams was supported by volunteers who had visited households to encourage eligible girls to attend for vaccination.

#### School records and attendance

In primary school records examined in these communities, the documented age range for girls in Class 4, the class being targeted for HPV vaccination, was 9–12 years. The situation assessment identified several potential challenges to a future school-based HPV vaccination programme including a reluctance by some government schools to provide access to their records, incompleteness of school data, missing attendance registers and missing or unknown date of birth or age information. Names in registers were often not separated by gender, making it time-consuming to confirm the number of girls in the target school year. It was also difficult to verify the number of girls who had enrolled but subsequently dropped out of government schools.

Absenteeism rates for female pupils ranged between 1–25% each day. This was higher at the start of term and, in Kajiado Country, on market days. Reasons for female absenteeism and school drop-out cited in FGDs and SSIs included girls being expected to contribute to the household income and to provide food for themselves and younger siblings. In Korogocho, other reasons included parental pressure for girls to sell alcohol and drugs, violence at home, or because girls were begging or engaging in sex work. In the pastoralist community, pregnancy and early marriage were reported as key reasons for leaving school as it was considered culturally unacceptable for girls to restart school following these life events. Additional reasons for missing or leaving school in Kajiado included recovery post-female genital mutilation (FGM), having to care for livestock and migrating with the family during the dry season in order to find water and grazing for livestock.

“…people are still keeping large stocks of animals and most of the children don’t go to school so that they can look after the animals.”

(Father, FGD Kajiado).

Long distances (up to 10km) to walk to school every day and concerns about encountering wildlife on the way were also cited as reasons for not attending school or for enrolling in school only when the girl was considered old enough to walk there safely.

The situation assessment found several interventions to encourage retention in schools including a school feeding programme, provision of free sanitary towels in schools, and peer tracing to identify girls who did not attend school. These were, however, not universally implemented in all schools in the communities and it was unclear how successful these had been.

#### Health care, treatment and vaccine beliefs

Korogocho had government, NGO-supported and private health facilities. Mashuru town had one government health centre and two private clinics; none of the participants reported using these latter clinics. Although most adult participants stated that they had attended government clinics to vaccinate their children because the service is free, reported barriers to health facility attendance were numerous and included distance to the health centre, complaints about the quality of care, a preference for traditional medicine, religious beliefs and, in Kajiado, seasonal features such as high rivers in the rainy season preventing access. CHWs were valued and mentioned in both sites as sources of information and as mobilizers for vaccination.

Participants in both sites believed that poor education about vaccines influenced vaccine uptake. There was, however, general support and trust in vaccines, with people seeing the benefits of vaccination, especially in children:

“The Maasai have started understanding the importance [of vaccines]. …. we have seen they have really helped a lot, because ....many children used to be born with polio but nowadays it is difficult to find children with polio.”

(Religious Leader, FGD Kajiado)

However vaccine mistrust was reported by a few participants in both communities.

“What are you vaccinating us against yet we are not sick? It seems that if you want to vaccinate us, there must be something you are gaining from that.”

(Community Leader, FGD Korogocho).

This was sometimes mistakenly related to non-governmental organization (NGO) efforts to stop the practice of FGM.

#### Sexual debut, sexual behavior and marriage in adolescent girls

Sexual debut was reported to occur at an early age in both communities, with reports of girls having sex as young as 8 years old and getting married as early as 10–13 years. In Korogocho, alcohol was seen as one facilitating factor for early sex.

“If the mum sells changaa [alcoholic brew] the drunkards come…. We don’t know at what age the men will start wanting to have sex with her, even at 8, 9 or 10…”

(Father, FGD Korogocho).

Some Korogocho community leaders also reported that early sexual activity in girls was facilitated by the media, the availability of pornography, such as DVDs, collected from Dandora dump and, in crowded households, by observing adults having sex.

“There is only a single room. …. even before the children go to sleep they have already started working (having sex), so the child in her head sees what is going on and on the following day when she goes out playing with her peers, she tries out what she saw at night. This makes our children start engaging in sex at an early age ….”

(Community leader, FGD Korogocho)

Early marriage or living ‘as married’ was reported by girls and parents in both sites, including arranged marriages, sometimes to older men, and unplanned pregnancy and poverty were also reported as other reasons for early marriage.“Those girls are given away for marriage in exchange for cows. This forces them to leave school early for marriage.....Sometimes they even take the girls out of school to go and look after the animals which opens more ways for them to meet the Morans (young warriors) whom they cannot avoid and then they get pregnant at an early age and are given away for marriage.” (Religious Leader, SSI Kajiado).

### Perceptions of a future HPV vaccination programme

#### Knowledge of cervical cancer, HPV and willingness to accept an HPV vaccine

Many of the participants had heard of cancer but, with the exception of health professionals, few participants had much detailed knowledge about cervical cancer. For those who had heard of it, cervical cancer was sometimes perceived as being related to sexual activity.

Among non-health professionals, few had any knowledge about HPV and the HPV vaccine and therefore participants were provided with information about HPV and HPV vaccine before the rest of the interview or discussion and then asked about their potential acceptance of the vaccine. Most participants were very supportive of an HPV vaccination programme.

“And now in our village, cervical cancer has become very rampant and some who get it don’t know what disease they are suffering from…. We will be holding meetings together and educate these girls so they can learn about this vaccine.”

(Community Leader, FGD Korogocho).

Further to this, most parents interviewed were willing for their daughters to be vaccinated.

“I am very grateful; I would even prefer it starting tomorrow.”

(Mother, FGD Kajiado).

Girl participants also expressed their willingness to be vaccinated.

There were conflicting views about the requirements for formal parental consent. Several teachers in Korogocho believed that parental consent should be required. Others believed that parental refusal should be challenged if the daughter wished to be vaccinated. One girl SSI participant stated that, if her parents refused to allow her to be vaccinated, she would report them to the police. Some teachers and religious leaders suggested that the chief should intervene if a parent did not permit his/her daughter to receive the vaccine.

“I think if the parent is just serious and doesn’t want the child to participate, we can refer that to the chief. Then from there the chief can follow it up.”

(Teacher, FGD Korogocho).

#### Potential barriers to vaccination

Inadequate information about HPV vaccine was seen as the most important potential barrier to the successful roll-out of a national programme.

“....I remember while I was in school we heard that people were coming to vaccinate pupils. The information was not well communicated, hence all the pupils ran away.”

(Religious Leader, FGD Korogocho).

This was a particular concern in Kajiado because of high illiteracy rates, reluctance by some to accept modern medicine, and suspicion that activities surrounding girls have to do with the frequently resented criminalization of FGM. Several participants drew parallels between community resistance to stopping FGM and the fact the HPV vaccine will be provided to the same age group of girls that undergo this procedure.

“Even though there is education about FGM, it is still practiced because there are those [parents] who don’t want to stop. Therefore you may find that, whilst a girl of age 11–13 who goes to school will be able to understand when she is told about cervical cancer vaccine, …her parent may think that the government has come up with another way to stop FGM.”

(Religious leader, FGD Kajiado).

A major concern was that rumours about potential effects on fertility would undermine vaccine uptake as it had done in the TT vaccination campaign.

“Maybe the government wants to do something for the girls so that they don’t have partners at school… They are being injected so that they don’t become pregnant at school.”

(Teacher, FGD Kajiado).

Provision of information about the vaccine and its benefits was felt to be critical to prevent this.

Injection pain, fear of side effects and the fact that the HPV vaccine would only be offered to girls were also mentioned as factors potentially affecting vaccine uptake.

“…the boys will enquire why it is only the girls who are getting the vaccine…The boys will discourage the girls, telling them that the vaccine will damage their lives.”

(Community Leader, FGD Korogocho).

Discrimination against boys was raised as an issue, especially by males. Boys in Kajiado stated that they would need clear information about why only girls will receive the vaccine and that it would be useful if boys were also taught about cervical cancer and HPV. One HW made the point that excluding boys from vaccination would not prevent transmission of the virus.

“Why target women and not men? Why don’t you target the source? Why don’t you put out the fire from where it starts.”

(District Public Health Nurse, Kajiado).

The fact that the vaccine should ideally be given before girls have passed sexual debut was seen as potentially discouraging young girls who have already had sex from getting the vaccine.

“If you say that only those who have not had sex should come, then many will go away.”

(Community leader, FGD Kajiado).

However, the concern that HPV vaccination might encourage girls to feel they are safe to engage in sex following vaccination was only mentioned by one participant.

Community leaders in Kajiado raised concerns about whether vaccination timing would be compatible with household duties or taking livestock out to graze. Participants in both sites also stated that it would be hard to vaccinate girls if they had to travel long distances to receive the vaccine and that girls who were pregnant or who married early would be out of school and would not have access to the vaccination.

There were also concerns about lack of parental guidance, particularly in Korogocho, which might lead to difficulties in obtaining parental acceptance for the vaccine if this was required.

The most significant barriers raised in the participatory ranking process for a hypothetical HPV programme varied by participant group but included similar factors as cited above, such as lack of adequate information about the vaccine, peer pressure, fear of injections, being unwell at the time of vaccination, being pregnant when vaccinations are offered, and religious and cultural factors (Table 2). Girls in Korogocho mentioned that menstruation around the time of vaccination could be a barrier (because they stay away from school), but ranked this fourth.

**Table 2:** Ranking of potential barriers and facilitators for a successful HPV vaccination programme.

Participants	Barriers	Facilitators
Teachers—Korogocho	1. Poor information	1. Creating awareness in the community
	2. Fear of painful injections	2. School-based vaccination
	3. Prolonged vaccination duration (e.g. boosters)	3. Involvement of teachers
	4. Lack of parental support	
	5. Religious beliefs	
	6. Negative peer influences	
	7. Lack of incentives	
Teachers—Kajiado	1. Poor information	1. Creating awareness in the community
	2. Distance to vaccination points	2. School-based vaccination
	3. Negative peer influence	3. Involvement of teachers
	4. Fear of being asked private questions	
	5. Fear of side effects and painful injections	
	6. Poor parental support due to illiteracy	
Boys—Korogocho	1. Negative peer pressure	1. Educating the community about the vaccine
	2. Ignorance	2. Vaccinating at places near their residences
	3. Lack of transport	3. Educating parents
	4. Lack of proper parental guidance	
Boys—Kajiado	1. Fear of the injection,	1. Offering vaccination near homes
	2. Negative peer influences	2. Educating community about the vaccine
	3. Poor parental support,	3. Educating parents
	4. Lack of information,	
	5. Distance and lack of transport	
Girls—Korogocho	1. Being sick at the time of vaccination	
	2. Fear of injections	
	3. Being pregnant	
	4. Menstruating at the time of vaccination	
	5. Long waiting time to get vaccinated	
	6. Balancing household chores	
	7. Ignorance	
	8. Already engaged in sexual activity from rape or otherwise	
Girls—Kajiado	1. Already pregnant	
	2. Lack of parental support	
	3. Being sick at the time of vaccination	
Community Leaders—Korogocho	1. Early pregnancy	1. Increased awareness
	2. Cultural/religious barriers	2. Involvement of all concerned
	3. Menstruating at the time of vaccination	
	4. Residential mobility	
	5. Ignorance	
Religious Leaders—Kajiado	1. Being unwell at the time of vaccination	1. Increased awareness
	2. Fear of injections	2. Involvement of community
	3. Early pregnancy	
	4. Migration	
	5. Poor communication	

#### Facilitating factors and strategies to achieve high coverage

Stakeholders suggested a number of strategies to overcome potential barriers. These included sensitising the community, especially to avoid rumours and myths, and developing strategies to reach out-of-school girls. A number of suggestions were given by interviewees and FGD participants. Government endorsement, provision of good information to all community members including boys and men since they can influence uptake of vaccine in girls, support from community leaders, cost free provision, and protection against cancer were all important factors in participants’ willingness to support HPV vaccination.

“The vaccine will be accepted since it has come from the government and they know the government cannot come up with something to destroy their children.”

(Community leader, SSI Korogocho).

It was suggested that information could be disseminated in several ways, including through the media (especially radio), posters, dramas, and incentives to get the vaccine (e.g. a free T-shirt), as well information disseminated by community leaders and village elders.

“Start with the village elders. These are the ones who have the loudest voice in the village.”

(Community leader, FGD Korogocho).

Chiefs, religious leaders and CHWs were suggested as being able to provide information about HPV vaccine because they were widely trusted in both communities and could deliver messages through churches and mosques, or through large meetings at a chief’s baraza (i.e. a meeting organised by a chief to inform the community). Parent meetings and meetings with girls were also considered very important.

Whilst participants understood that, under the national guidelines, boys would not be eligible for the HPV vaccination since the vaccine is being offered to girls in order to prevent cervical cancer, a few participants suggested that boys should receive some health intervention at the same time. It was also stressed that efforts should be made to avoid myths and rumours about the vaccine and that the MOH should work closely with religious leaders and chiefs to provide information to counter potential rumours around infertility.

To minimise school absenteeism, participants suggested involving school teachers to ensure that girls attend school on the specified vaccination date, and one stakeholder suggested running the HPV vaccine programme alongside the de-worming programme since both parents and girls are familiar with this intervention. Provision of vaccination cards to girls and providing information on dates for follow up doses to teachers and parents and announcements through the media and other channels were considered important factor to facilitate completion of the vaccine course.

Home visits to out-of-school or absent girls were mentioned to deal with girls who missed doses. Key people suggested for this task included chiefs, headmen, teachers, school-based social workers or CHWs. No specific strategies were suggested in order to reach girls who had migrated with cattle on vaccination days except to try and follow girls at chief’s camps or on market days. Mobile clinic services were considered important to bring services close to where people live.

The main facilitating factors mentioned in the participatory ranking exercise were creating awareness and educating communities about the vaccine, offering vaccines near homes and offering school-based vaccination.

#### Potential venues for vaccinating girls in these communities

Most stakeholders and other participants were supportive of HPV vaccination being conducted at schools because of the ability to vaccinate a large number of girls quickly, trust in teachers’ authority, and lack of fear about attending a school. Schools were sometimes also considered to be more easily accessible than health facilities.

“For those children that live far, maybe leaving the school and going to a health facility will be hard. If it is in school, just do it there.”

(Mother, FGD Korogocho).

Both boys and girls in Kajiado and girls who worked on the Dandora dump were positive about vaccines being offered at local schools.

“We don’t mind. As long as we are in our Sunday best, we will get the vaccine. We go back home, change and wash, then go to the site.”

(Girl, FGD Korogocho).

Health facility vaccine provision was considered useful to potentially vaccinate some out-of-school girls, especially in Korogocho. Other potential venues for vaccination included watering points, markets, chiefs’ camps, and churches. Religious leaders suggested that conducting vaccinations at chiefs’ camps might reach more girls.

“If it is done by the school or church, you run the risk of getting only the people involved in that school or church. If you do it at the chief’s camp, you know it’s for the whole community.”

(Religious Leader, SSI Korogocho).

The important point made by participants was that the vaccinations should be conducted within the community and that the vaccination venue should be accessible. Offering the vaccine through many centres as feasible in Kajiado County was also felt to be an important strategy to reach as many girls as possible.

Using a 3-day vaccination campaign approach was suggested by some parents who were probably familiar with other vaccine campaigns. There was also a suggestion that specific outreach activities would also be needed to reach girls in Maasai communities. However, this approach was not supported by the health workers in Kajiado, mainly because they felt they would not have the staff to manage such an approach and that using a school venue for vaccination would ensure high coverage.

“If you go to school you are able to get a big number at one place so it is easy to administer to them and finish as opposed to moving from house to house, or village to village.”

(Public Health Nurse, SSI Kajiado).

MOH staff recognized that reaching these communities with health interventions is challenging. Service delivery has relied on a combination of static clinics along the routes that pastoralists frequently travel, outreach services and mobile clinics (using e.g. using motor vehicles, motor cycles, bicycles and camels). Given that these systems are in place, HPV vaccination could also potentially be integrated or delivered with these other services.

## Discussion

This is the first study to examine issues around access and attitudes to HPV vaccine in hard-to-reach communities. Nairobi slums and Maasai nomadic pastoralist communities represent populations that may be difficult to reach for an adolescent/pre-adolescent health intervention for a number of reasons, including mobility, difficulty accessing communities to offer services, availability to receive vaccine, school absenteeism and drop-out, and early age of sex and marriage[11–14]. Despite this, and, in common with other sub-Saharan African populations[15], a general lack of knowledge and awareness about cervical cancer and the HPV vaccine, there was considerable interest in receiving HPV vaccination in these communities, with many participants indicating they would support vaccination if adequate and appropriate information was available and efforts were made to address concerns and rumours. Encouragingly, this potential acceptance also included very hard-to-reach girls, such as those working in the rubbish dump at Dandora.

Lack of knowledge about cervical cancer and HPV has been seen in many other populations in sub-Saharan Africa, including in previous studies of women in Kenya attending health services[16–19]. It was clear from the results that extensive social mobilisation, with provision of adequate information about the benefits of vaccination, will be key to ensuring uptake of vaccine.

Potential barriers to vaccination included concerns that have been previously reported in other studies, such as rumours around infertility and lack of information about the vaccine[19–21]. Offering a female-only vaccine, concern about vaccination being linked to FGM prevention activities, and potential male discrimination were also mentioned as possible barriers by Maasai participants. This may be more of an issue in these more traditional communities but several of these concerns were mentioned by some non-pastoralist participants in Tanzania prior to a vaccination pilot project[19]. This was however, not a major reason for girls not accepting vaccine in Tanzania once vaccination actually commenced[22,23], possibly because social mobilisation and discussions with HW had addressed these issues. Concerns were expressed about emphasising that the vaccine should be offered to girls who had not had sex since it was felt that this would discourage young girls who are eligible for vaccination to be vaccinated if they had passed their sexual debut. This may be particularly important in setting such as slums and pastoralist communities where sexual debut for some girls may be very early.

There was a general consensus that school-based vaccination would be the most efficient approach, even in these settings, but that this would need to be supplemented with provision of additional vaccination venues close to where out-of-school girls work or live that might include mobile out-reach activities. Relying on health facility-based vaccination alone would probably not be successful, especially in remote pastoralist areas where health facilities may be difficult to access and may not be the first place people go when seeking treatment for medical problems.

It is hoped that these data will inform development of an appropriate design for a future vaccination programme, and development of effective and acceptable advocacy, communication and social mobilization strategies for the programme. To this end we have several recommendations.

The vaccine should be offered to girls as young as possible (9–10 years), given the early age of sexual debut and marriage in these hard-to-reach communities. While efforts to postpone sexual debut need to be put in place, information about the vaccine should not emphasise targeting girls before their sexual debut so that young sexually-experienced girls who may be at particularly high risk of HPV, including young married girls, are not discouraged from getting the vaccine. Emphasis should be made on the vaccine’s value in preventing cervical cancer to the girls once they become adults. This approach will increase acceptance of the vaccine in these populations. Effective community mobilisation and sensitisation will require involvement of many individuals besides health workers, teachers and chiefs. Messages may need to be delivered at wider venues, such as chief’s barazas. The use of CHWs, who have been advocated as providing a sustainable approach to provision of health care in pastoralist regions[24], and religious and community leaders to debunk myths and misconceptions about the vaccines will increase acceptance. Given the experiences with TT vaccine and beliefs around modern medicine, discussions and provision of information to religious leaders will be important to avoid the spread of negative rumours or misinformation. Finally, consideration of a campaign approach in multiple venues for the first dose of HPV vaccine may be useful, especially in pastoralist communities if this is financially feasible, with mop-up activities for those girls who have temporarily migrated in pastoralist areas or who could not afford to miss a day of activities to support themselves. Offering HPV vaccine alongside other well-run and highly acceptable adolescent/school-based interventions may be helpful if these interventions are in place. By bringing the education and health sectors together, HPV vaccination may provide a platform for other information and services such as the need for girls to enrol and complete school including return to school for pregnant girls after childbirth, the harm that early marriage and FGM cause to girls, the value of hygiene and sanitation, the need for screening for cervical cancer for their mothers, and so on. However, it will important to evaluate carefully whether any of these parallel interventions would actually decrease HPV vaccination coverage. For example, linking HPV vaccination to education against FGM might discourage parents of girls who support FGM from allowing their daughters to be vaccinated.
